# Large Submucosal Uterine Leiomyoma Prolapse Into the Vagina After Complicating a Pregnancy: A Case Report

**DOI:** 10.7759/cureus.49116

**Published:** 2023-11-20

**Authors:** Moayad S Almusaylim, Faten A Darwish, Fatimah A Alahmad, Mustafa A Alsaleh, Montadhar M Almohammedsaleh

**Affiliations:** 1 Obstetrics and Gynaecology, College of Medicine, Imam Abdulrahman Bin Faisal University, Dammam, SAU; 2 Obstetrics and Gynaecology, King Faisal General Hospital, Al-Ahsa, SAU; 3 General Surgery, College of Medicine, Imam Abdulrahman Bin Faisal University, Dammam, SAU

**Keywords:** prolapsed fibroids in pregnancy, prolapsed submucosal uterine fibroids, hysteroscopic myomectomy in pregnancy, pregnancy complications, incomplete abortion, uterine fibroids in pregnancy, multiple uterine fibroids

## Abstract

Uterine leiomyomas are the most common pelvic neoplasm in females. They are non-cancerous monoclonal tumors that develop from the fibroblasts and smooth muscle cells of the myometrium. They can develop in females of reproductive age and post-menopausal as well. When symptomatic, they frequently manifest as abnormal uterine bleeding and/or pelvic pain or pressure. Reproductive effects are also possible in fibroids such as infertility and poor pregnancy outcomes. In this report, we present a case of a 39-year-old woman, G9P4A4, GA 16 weeks who came to the ER with an incomplete abortion at home after which she had large multiple uterine submucosal fibroids prolapsing into the vagina. On ultrasound, the placenta was still inside, and a large submucosal anterior uterine fibroid and a posterior uterine fibroid were found with sizes 10x10 cm and 2x3, respectively. Evacuation and curettage (E&C) could not be completed because fibroids were obstructing and limiting the access. The patient was managed medically and then discharged, after which she came back with prolapsing uterine fibroids and part of the placenta. In the end, she was managed by hysteroscopic myomectomy.

## Introduction

Uterine leiomyomas are the most common benign smooth muscle tumors of the genital tract in women between 35 and 40 years old [1,2]. Depending on the trimester of assessment, the reported prevalence of uterine leiomyomas in pregnancy ranges from 1.6% to 10.7% [3,4]. Classifications of leiomyomas depend on their location in the uterus as described in the International Federation of Gynecology and Obstetrics (FIGO) system for fibroid as follows: Subserosal myomas, intramural myomas, cervical myomas, and submucosal myomas which can protrude into the uterine cavity similarly to the case we present in this report [5]. The possible effects of fibroids on pregnancy and the possible effects of pregnancy on fibroids are frequently expressed since these tumors are more common in females of reproductive age. The complications related to leiomyomas depend on the site, size, and number; however, the majority of patients do not experience any complications related to leiomyomas during pregnancy. The most common problem is pain, and there may be a slightly increased risk of obstetric complications, such as preterm labor and birth, fetal malpresentation, placental abruption, and early pregnancy loss, especially if they have multiple fibroids, retroplacental fibroids, and size larger than 5 cm [6]. Other complications of leiomyomas are determined by their size and location, including abdominal pain, abnormal uterine bleeding, increased abdominal girth, urinary frequency, and infertility [7]. Thirty percent of all uterine fibroids in perimenopausal women are asymptomatic, slow-growing fibroids [8]. Herein, in this article, we report a case of large multiple uterine submucosal fibroids prolapsing into the vagina, the effect on pregnancy, and how it was managed.

## Case presentation

A 39-year-old woman, G9P4A4, GA 16 weeks presented to the emergency room (ER). Her past medical history was unremarkable apart from sickle cell trait. Her past surgical history was significant for three times evacuation and curettage (E&C) and not known to have leiomyoma previously. The patient presented to the emergency department aborting the fetus at home with minimal bleeding on Friday with no other complaint. The patient arrived at the ER in good overall condition and with alert consciousness. The following vital signs were present: oxygen saturation on room air of 98%, blood pressure of 128/78 mmHg, heart rate of 88 beats per minute, respiration rate of 18 breaths per minute, and temperature of 36.5°C. The fundal level of the uterus showed the presence of a mass as high as the umbilicus. Vaginal examination OS opened with mild PV bleeding. On ultrasound, the placenta was still inside, and a huge anterior uterine fibroid and a posterior uterine fibroid were found with the size 10x10 cm and 2x3, respectively.

Management

**Figure 1:**
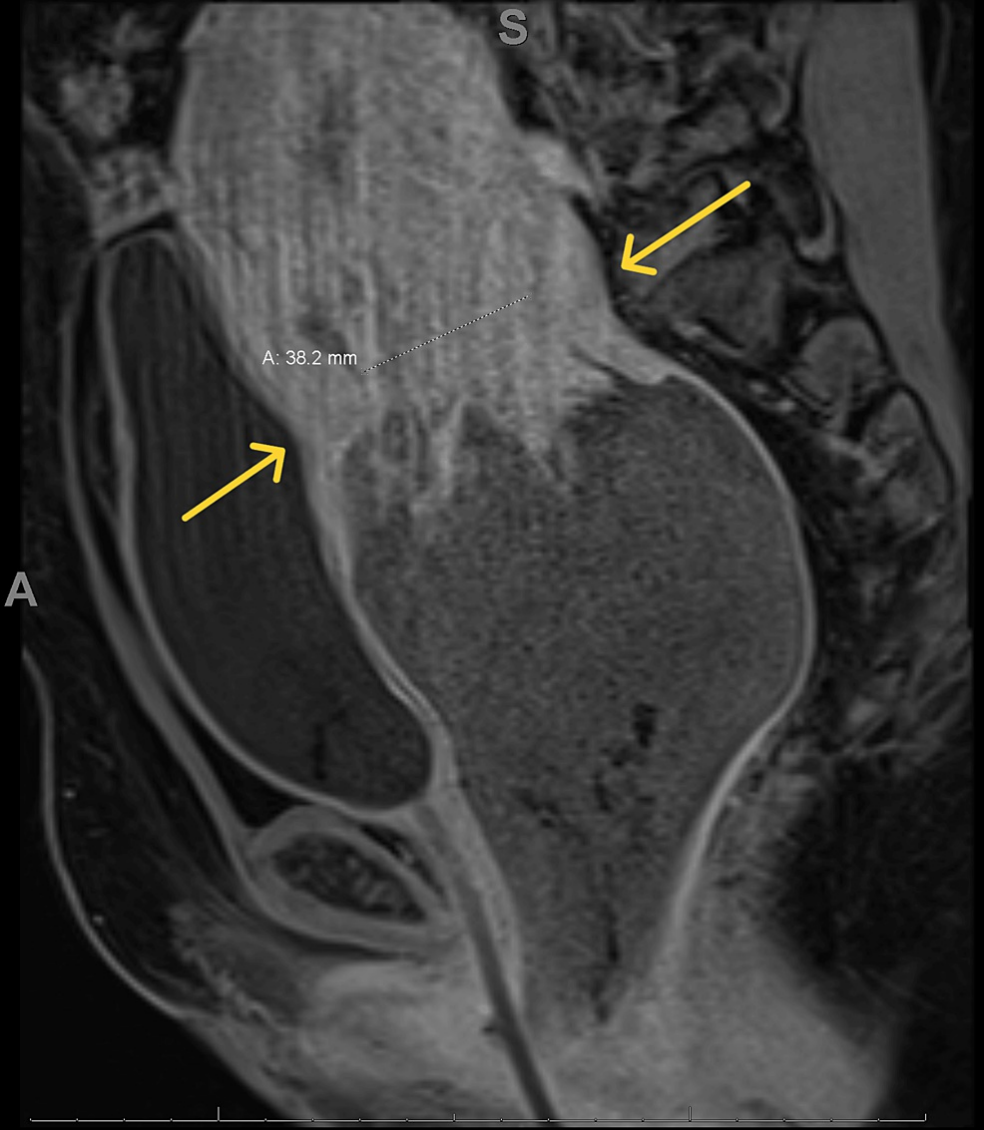
There is oval shape mass prolapsing within the vagina and attached to the posterior uterine wall. It measured around 10*9.5*11.2 cm (AP*TR*CC), showing no contrast enhancement indicating ischemia.

**Figure 2:**
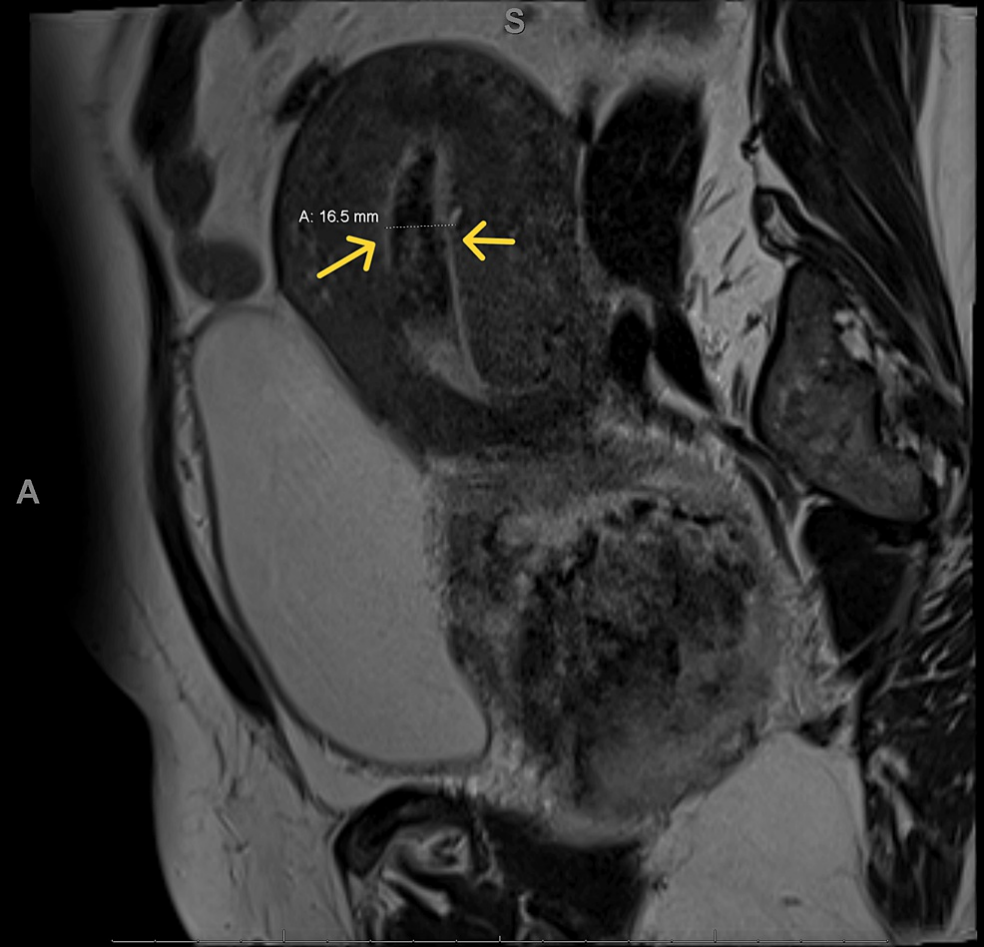
There is thickening of the endometrium measuring 1.6 cm containing soft tissue components which represents retained products of pregnancy.

The patient was diagnosed with a subinvoluted uterus that retained products of conception, with multiple submucosal leiomyomas. She was planned to be managed with E&C, but it could not be completed due to large uterine fibroids which were obstructing and limiting access. The patient had vaginal bleeding and was admitted to the ward for two days. She was managed by metronidazole 500 milligram PO Q2hr/wk, piperacillin/tazobactam 4.5 gram Q6H for seven days, and misoprostol 400 microgram three doses Q3hrs. However, she did not pass anything. She was consulted for medical and surgical management. She was discharged on expectant management and told to come to the hospital whenever she passed anything. After five days, she came to the ER complaining of bleeding and prolapsing tissue into the vagina, speculum and point-of-care ultrasonography were done which found part of the placenta and uterine fibroids were prolapsing through the cervical canal into the vagina. Manual removal of the placenta under anesthesia was tried but failed due to the difficulty of removal. MRI was done and showed the following findings: First, a prolapsed uterine fibroid was seen in the vagina and attached to the posterior wall with no enhancement indicating ischemia (Figure 1) and second, thickening of the endometrium with enhancing content which represents retained products of pregnancy (Figure 2).

One day after, the patient underwent hysteroscopic myomectomy with removal of leiomyoma and placenta piece by piece. The patient was monitored in the ward for five days post-operative because she had signs of infection for which she received medical management consisting of amoxicillin/clavulanic acid, metronidazole, and gentamycin. The patient was discharged after stabilizing her at 11:30 a.m. on Sunday 2/7/2023.

Specimens of removed tissue were sent for histopathological examination and showed the following: The posterior wall leiomyoma specimen reports partially autolyzed fragments of interlacing facia of smooth muscle fibers, with focal macrophages. The morphologic features are compatible with leiomyoma. The pathology examination of the placenta tissue reports uterine tissue consisting of chorionic villi, gestational endometrium, and decidua.

## Discussion

Leiomyoma's etiology is mostly unknown, although it is known that they respond to progesterone and estrogen stimulation by growing and that their prevalence rises during the reproductive years and sharply declines after menopause. In fibroids compared to healthy myometrial tissue, higher levels of aromatase, estrogen, and progesterone receptors have been found. Obesity, pregnancy, early menarche, and exposure to exogenous estrogen typically have an effect on fibroid growth [3,9]. Changes in estrogen and progesterone levels, uterine blood flow, and, possibly, human chorionic gonadotropin levels can all have an impact on the fibroid size. The fibroid size can also be affected by maternal characteristics such as age, race or ethnicity, parity, and miscarriage history [10]. About 90 percent of patients with fibroids discovered in the first trimester will have a decline in total fibroid size when investigated again three to six months postpartum, but 10 percent will have an increase in the size [11]. Patients who are on progestin-only contraception may experience less regression. It has also been proposed that the pathophysiology of uterine fibroids includes a genetic component. Chromosomes 6, 7, 12, and 14 have high-frequency alterations that have been observed in uterine leiomyomas [9]. Other stimulations are growth factors such as insulin-like growth factors and transforming growth factor-beta [7]. During pregnancy, uterine fibroids are usually asymptomatic, with the exception of rare possible red degeneration that may present with acute abdomen. Individuals with symptoms could experience pain, pelvic pressure like frequency or urgency in urination, and/or vaginal bleeding. The most frequent symptom is pain [12,13].

Most pregnant patients with fibroids do not experience any complications during pregnancy related to the fibroids [14]. Painful deterioration is the most frequent complication when it does occur, it is worth mentioning that the gold standard treatment for painful degeneration is conservative treatment which includes admission to the hospital, analgesics, IV fluids, and antiemetics [15]. Although all studies do not demonstrate a higher risk of adverse events, there also appears to be a slightly increased risk of complications such as preterm labor and birth, early pregnancy loss, placental abruption, and fetal malpresentation [6]. This is what makes this case distinctive -besides the fact that the fibroid was too large and prolapsing into the vagina- the patient developed complications related to leiomyoma in the form of bleeding and pregnancy loss, it is important to note that her previous pregnancy loses are not related to her current condition. In some patients, submucosal fibroids -like in the presented case- appear to affect implantation, placentation, and ongoing pregnancy. Intramural fibroid's effects remain controversial. On the other hand, fibroids that are primarily subserosal or pedunculated are less likely to result in early pregnancy loss. The presence of multiple fibroids may increase the risk of miscarriage [16].

Diagnosis depends mainly on pelvic examination, and pelvic ultrasound, in addition to color Doppler with TVS. This diagnosis could be supported by characteristic symptoms, the most common of which are menorrhagia, pelvic pain, and subfertility. Although pathological evaluation is considered to be the definitive diagnosis, it is preserved for cases in which the uterine mass is considered to be a precancer or cancer. Physical examination, specifically pelvic bimanual examination, provides some vital information that helps in differential diagnoses and monitoring the size of the fibroid. A fixed uterus raises malignancy suspicious which leads to the need for more thorough investigations [16].

Imaging studies in uterine fibroid patients are organized into two steps. Step one is pelvic ultrasound which has the feature of minimally invasive and the ability to visualize the genital tract, this modality has high sensitivity but has a low ability to differentiate between benign and malignant masses. Step two specifies when complex intervention of malignancy is suggested, it includes contrast-enhanced ultrasound and magnetic resonance imaging (MRI) which are considered the most effective modality to evaluate the size and location of the fibroid. Although MRI is considered the gold standard, it is the last resort due to its expenses [17,18].

The European Society for Gynecological Endoscopy classification describes submucosal leiomyomas in three levels: level 0 = completely in the uterine cavity; level 1 = with its largest portion inside the uterine cavity; and level 2 = with its smallest portion in the uterine cavity [19]. In general, Hysteroscopic myomectomy is the best treatment option for patients who suffer from heavy menstrual bleeding caused by submucosal fibroids, due to the minimally invasive and fertility preservation of fertility [16].

Management of leiomyoma during pregnancy can be as follows: antepartum transvaginal myomectomy, unless there is an easily accessible pedunculated leiomyoma on a slender stalk, it is typically advised against removing prolapsed fibroids in pregnancy because the dangers almost certainly outweigh the benefits. Removal may result in membrane rupture, hemorrhage, or/and pregnancy loss. Myomectomy of pedunculated or subserosal fibroids has infrequently been performed antepartum for the treatment of acute abdomen or obstruction, and myomectomy may be necessary during cesarean delivery in order to close the hysterotomy [20]. Preconception myomectomy decisions are decided on a case-by-case basis and depend on several factors, such as the patient's age, past reproductive history, the severity of their symptoms, the size, and the location of their fibroids.

Finally, there are insufficient data to advise a minimal time interval between myomectomy and conception [21].

## Conclusions

Although uterine fibroids rarely complicate pregnancy, they may cause several complications including pregnancy loss like in this case report. Furthermore, large multiple submucosal uterine fibroids that are prolapsing into the vagina may lead to difficulty in performing E&C which makes hysteroscopic myomectomy necessary to perform E&C successfully.
